# The inhibitory impact of collaboration on the continued influence effect of misinformation

**DOI:** 10.3389/fpsyg.2024.1487146

**Published:** 2024-10-25

**Authors:** Gongxiang Chen, Yuxuan Zhong, Sujie Li

**Affiliations:** ^1^School of Education and Psychology, University of Jinan, Jinan, China; ^2^Office of Student Affairs, University of Jinan, Jinan, China

**Keywords:** CIE, cross-cuing, free collaboration, re-exposure, turn-taking collaboration

## Abstract

The continued influence effect (CIE) of misinformation refers to the persistence of misinformation’s impact on memory and inference even when individuals are aware of a retraction. This study examined whether collaborative processes affect the CIE and investigated the underlying mechanisms through three experiments. Experiment 1 explored the general impact of collaboration on the CIE. Experiment 2 further dissected collaboration into turn-taking and free collaboration conditions, assessing their effects on the CIE at various recall intervals. Building on these findings, Experiment 3 delved into the mechanisms driving the differential effects of turn-taking and free collaboration on misinformation correction. Results revealed that turn-taking collaboration consistently mitigates the CIE, while the effect of free collaboration on misinformation correction is moderated by recall time. This variation is attributed to differences in re-exposure, cross-cuing, and forgetting across collaboration types. The present study contributes empirical support to the Knowledge Revision Theory of the CIE.

## Introduction

Misinformation is prevalent in our everyday lives and refers to information initially accepted as true but later publicly retracted or corrected. Misinformation poses great challenges to human cognition and social development. In real life, there are some misinformation accompanied by sudden and emergency events, such as SARS virus and the novel coronavirus pneumonia epidemic. Despite these corrections, the erroneous information often continues to impact individuals’ memories and factual judgments, thereby influencing their decisions (Brydges et al., 2018; Swire-Thompson et al., 2023). This is called the continued influence effect of misinformation (CIE; Chan et al., 2017; Koetke et al., 2023; Koo et al., 2021). In the United Kingdom, a 1998 study suggesting a link between a common childhood vaccine and autism generated considerable fear in the general public concerning the safety of the vaccine. The UK Department of Health and several other health organizations immediately pointed to the lack of evidence for such claims and urged parents not to reject the vaccine. The media subsequently widely reported that none of the original claims had been substantiated. Nonetheless, in 2002, between 20 and 25% of the public continued to believe in the vaccineautism link, and a further 39–53% continued to believe there was equal evidence on both sides of the debate (Hargreaves et al., 2003).

The impact of collaborative recall on the continued influence effect (CIE) has not yet been thoroughly explored by researchers. Existing studies have predominantly focused on objective factors such as the source of information, methods of correcting misinformation, and the presentation modes of misinformation, as well as participative factors including cognitive preferences, initial attitudes, levels of self-confidence, and cognitive abilities (Capraro and Celadin, 2023; DeVerna et al., 2022; Susmann and Wegener, 2023). Misinformation is a significant social issue, with its spread and correction often relying on interpersonal communication and collaboration (Butler et al., 2022). Research has indicated that collaborative processes can influence individual memory (Congleton and Rajaram, 2011; Wissman, 2020). Specifically, when misinformation is corrected—referred to as the original misinformation—collaboration might affect how individuals remember both the original misinformation and the corrected information. This can influence the strength of memory encoding and subsequently impact the continued influence effect (CIE). Accordingly, this study examined whether collaboration affects the CIE, investigating whether it facilitates or inhibits this effect. Additionally, the study explored how different collaboration conditions and recall intervals influence the CIE and examined the underlying mechanisms driving these varying impacts.

### The continued influence effect of misinformation

The paradigm of CIE was originally developed by Wilkes and Leatherbarrow (1988), has been continuously used (Susmann and Wegener, 2023), in which participants first read a story with misinformation, such as a warehouse fire, and were led to believe that there might be flammable material in the wardrobe, which was the cause of the fire when the material was presented. Participants in the correction group received corrections for the misinformation they had read (i.e., they were informed that the wardrobe was empty), and no further corrections were provided to the non-correction group. Finally, the participants were asked to complete a Discrepancy Detection Test, in which they recalled the content of the material they read and inference about the cause of the warehouse fire.

Kendeou et al. (2014) proposed the Knowledge Revision Theory to explore the mechanisms inherent in the continued influence effect of misinformation, including five principles: encoding, passive activation, coactivation, integration, and competing activation (Kendeou et al., 2013). The encoding principle states that once information is present (correct or incorrect information) it is first encoded into memory; the passive activation principle states that all information is passively activated, independent of whether the activated information is correct or relevant, and the coactivation principle is an outgrowth of both the encoding and passive activation principles and is how new information is interconnected with the previously acquired information. The integration principle is the basis for knowledge correction, where new information will begin to dominate the entire information network as the amount of newly acquired correct information continues to increase; finally, the activation of corrected information competes with the activation of previously incorrect information for the final output (competitive activation principle). Several works have demonstrated that CIE is partly driven by information familiarity (Ecker et al., 2017). Throughout the corrective process, misinformation is frequently repeated, which increases its encoding strength and familiarity. As a result, CIE is improved since familiar claims are more likely to be believed (Bailey et al., 2021). Consistent with this logic, repeatedly presenting corrected information enhances its encoding and improves individuals’ memory of the correction, making it seem more familiar and more likely to be accepted (Ecker et al., 2014). Consequently, the activation of the corrected information competes with that of the original misinformation, as the repetition influences the strength of encoding.

### Collaborative memory

When two or more group members work together to recognize, retain, and recall what they have learned, the process is called collaborative memory (Basden et al., 2000; Wissman, 2020). A typical paradigm of collaborative memory consists of three phases (Wissman, 2020): the learning phase, the interference phase, and the recall phase. In the learning phase, all participants individually learned the study material, followed by the interference phase. Finally, there was a recall phase where participants were divided into collaborative and nominal groups. The participants in the collaborative group were divided into groups of three and worked together to recall the material they had learned. The members of the collaborative group were encouraged to freely discuss with each other and recall as many items as possible. The participants in the nominal group were tested individually for recall. The effect of collaboration on memory was investigated by comparing the number of items recalled in the recall test between the collaborative group and the nominal group. Many studies have subsequently incorporated one or more individual tests following the initial collaborative memory procedure to examine if collaboration has a lasting effect on subsequent individual memory (Congleton and Rajaram, 2011; Jalbert et al., 2021).

There is a large body of studies suggesting that collaborative recall tends to result in higher memory accuracy compared to nominal groups, known as post-collaborative memory facilitation (Rossi-Arnaud et al., 2020). Post-collaborative memory facilitation is thought to primarily involve two cognitive processes: re-exposure and cross-cuing (Congleton and Rajaram, 2011). Re-exposure refers to the opportunity for participants to relearn during collaborative recall when they hear someone else report items that they were unable to recall when alone (Mundorf et al., 2021). In addition, cross-cuing occurs when a person hears other group members recalling information that they have forgotten and uses this as a cue to recall additional information (Guynn, 2024).

Prior researches have categorized collaboration conditions as turn-taking collaboration and free collaboration (Abel and Bäuml, 2020; Wright and Klumpp, 2004). Turn-taking collaborative recall involves each person taking turn reporting one piece of information at a time, moving to the next person when a person reports no recall, and so on (Nie and Guo, 2023). The free collaborative recall involves participants as a whole recalling as many of the items they have just learned as possible, with free consultation, discussion, and reminders among group members. Some studies have found significant differences in the effects of various collaborative conditions on memory (Rajaram et al., 2020). However, the results of the current study are inconsistent. For example, Thorley and Dewhurst (2007) found that turn-taking collaborative groups demonstrated improved memory after collaboration, and there was no significant difference in recall outcomes between groups that free collaboration group and nominal group. Weihai et al. (2015) found that both turn-taking collaboration and free collaboration resulted in improved memory after collaboration. Based on previous contradictory findings, various collaboration conditions have varying effects on memory.

At the same time, we found that the time of collaborative recall, as set by previous studies under different collaboration conditions, is inconsistent. The time of recall directly affects the encoding strength of memory (Mundorf et al., 2021), which may be a factor influencing the varying degree to which different collaboration conditions affect memory. Most studies on collaborative memory do not have an explicit time limit. For example, Abel and Bäuml’s (2020) study, where the recall phase was not explicitly time-limited free collaboration was used, and social interaction was found to enhance memory. Numerous studies have also demonstrated this phenomenon (Harris et al., 2012; Bärthel et al., 2017). In contrast, another one utilized a free collaboration condition, allowing participants 2 min to collaboratively recall a scene. The study found that the recall scores of the free collaboration group were not significantly different from those of the individual group in an immediate recall test (Huff et al., 2016). In addition, Abel and Bäuml (2020) explored the effects of turn-taking collaboration on memory and found that the turn-taking collaboration group did not show post-collaboration memory facilitation when there were no time constraints (Wright and Klumpp, 2004). Weihai et al. (2015) set the recall time to 8 min for free and turn-taking collaboration groups and 4 min for individual groups and found that both turn-taking and free recall produced post-collaboration memory facilitation, and the amount of post-collaboration memory facilitation for turn-taking recall was higher than that for free recall. From previous studies, we know that the recall time in different collaborative conditions affects memory results. Therefore, it is necessary to investigate the impact of recall time on collaborative memory.

### The present study

This study intends to investigate the effect of collaboration on CIE. To explore this phenomenon, not only because it is common in daily life, but also because it is conducive to answering two important theoretical questions: (1) Does collaboration have an impact on CIE? Is this influence collaborative or facilitate? (2) What are the potential mechanisms by which collaboration affects CIE? This study intends to answer the above questions through three experiments. Experiment 1 provides two collaboration conditions, the nominal group and the collaborative group, to explore whether and how collaboration affects an individual’s CIE. Experiment 2 specifically investigated the effect of different collaboration styles and recall time on CIE. On the basis of experiment 2, experiment 3 explores the reasons for the different effects of different collaboration methods on CIE.

According to knowledge revision theory, the strength of memory encoding influences CIE, with the encoding activation of corrected information competing with the encoding activation of original misinformation for the final output (Newman et al., 2022). Numerous studies have shown that collaboration enhances individuals’ memory, as demonstrated by post-collaboration memory facilitation (Congleton and Rajaram, 2011; Wissman, 2020). When misinformation is corrected, collaboration may strengthen an individual’s memory encoding of the corrected information, thus enhancing memory and inference, and inhibiting CIE (Sanderson et al., 2022). Therefore, Hypothesis 1 of the present study is proposed: Collaboration inhibits CIE. Previous studies have divided cooperation into free cooperation and turn-taking cooperation, and different cooperation conditions have different effects on memory (Weihai et al., 2015). Moreover, previous studies did not consider the recall time, a variable that affects the intensity of memory coding. The influence of different cooperation conditions on CIE may be regulated by recall time. Therefore, Hypothesis 2 of the present study proposes that free collaboration and turn-taking collaboration inhibit CIE differently at different recall time. The variation in the degree of inhibition of CIE by collaboration conditions under different recall time may be attributed to differences in re-exposure, cross-cuing, and information forgetting during collaboration. If group members refer to the corrected information and its encoding strength increases, this leads to a greater acceptance of the correction by the individual and ultimately inhibits CIE. In addition, if group members are influenced by cross-cuing and refer to the corrected information, they can enhance the individual’s fluency in processing the corrected information and use it as a valid cue for extracting the information. This enhances the memory representation of the corrected information and leads to CIE inhibitory. Forgetting information, on the other hand, reduces the extent of memory encoding and facilitates CIE. Therefore, Hypothesis 3 of this study proposes that the various collaboration conditions exert varying degrees of influence on CIE at different time due to re-exposure, cross-cuing, and forgetting during the collaboration process. To test these hypotheses, the current study investigated the impact of collaboration on the continued influence effect (CIE) through a series of three experiments.

## Experiment 1

Experiment 1 aimed to explore whether collaboration impacts the continued influence effect of misinformation (CIE), specifically, whether it facilitates or inhibits CIE.

### Method

The experimental design was a 2 × 2 mixed design, with one between-participants factor and one within-participants factor. The between-participants factor was collaboration condition, which included two levels: nominal group and free collaboration group. The within-participants factor was correction condition, with two levels: corrected and uncorrected.

### Participant

An *a priori* power analysis in G*Power (Faul et al., 2007) suggested that we would need at least 36 participants to detect a high effect using ANOVA (*f* = 0.25; power = 0.95; *α* = 0.05). We therefore recruited 52 participants from an academic institution. As pre-registered, we removed those who failed the attention check (*n* = 4). This left a final sample of 48 participants (29 female, 19 males; Mage = 20.33, SDage = 1.47). All experiments conducted in this study were approved by the Ethics Committee of University of Jinan.

### Materials

#### Reading materials

The reading materials consisted of short newspaper-style articles written by Ecker et al. (2017). There were four scenarios, with two articles for each scenario. Additionally, there were two versions of the second article, which were evaluated by two psychology professionals to ensure they were well-written and aligned with the reading preferences of our national population. The first article, which was identical in all conditions, contained crucial information regarding the cause of the event. E.g. Early evidence suggests that the fire was intentionally ignited. There were two versions of the second article in each scenario, and each group of three people read the same version. The second article in the no-correction condition was only appended to the first article. E.g. The estimated burned area was approximately 50,000 hectares. The second article, in the corrected condition, included updated information in the additional description that contradicted crucial information presented in the first article. E.g. After conducting a comprehensive investigation and review of witness reports, authorities concluded that the fires were caused by lightning strikes (see Supplementary material for details).

#### Recall test materials

The comprehension questionnaire utilizes a modified version of the questionnaire employed by Xu et al. (2020). This questionnaire is used to measure CIE scores, which are specifically categorized into general memory, critical memory, corrected memory, and inference scores. The general memory score represents the participant’s overall recollection of the event. The critical memory score reflects the memory of the original misinformation. The corrected memory score indicates the memory of the reason for the most recent correction. Lastly, the inference score measures the extent to which the individual relies on the original misinformation for reasoning. Higher scores in general memory, critical memory, and corrected memory indicate a better level of memory, while lower reasoning scores indicate more rational reasoning. The questionnaire has specific scoring details, which can be found in the Supplementary material.

#### Interference materials

The interference material consisted of a 2-min multiplication and division exercise.

### Procedure

This Experiment combines the collaborative memory paradigm with the CIE paradigm. Participants were randomly assigned to either collaborative or nominal groups of three.

1. Learning phase: Three group members individually read pairs of short newspaper-style articles from each of the four scenarios. Each article was time-limited to 0.35 s per word. The order of the four scenarios was randomly presented in different groups. To minimize the chance of participants being reminded of the corrections, the order of the correction condition of the second article was consistently presented in a crosswise manner. The Experiment materials and order presented to members of each group were consistent.
2. Interference phase: All participants were required to complete a 2-min number count after reading the article. The participants were asked to complete some simple calculation tasks, such as 23× 5,128 ÷8, etc.
3. Recall phase: Participants were provided with a test paper. “Please write down any information you can recall from the four scenes.” Participants completed the test individually. Free collaboration Group: “Please write down any information you can recall from the four scenes after discussion and negotiation.” Three people can consult each other, discuss, and remind each other. If there are any controversial items, the group members can determine the trade-offs through discussion. Finally, the answers should be written down after a negotiation involving all three people. The recall time for both groups of participants is unlimited.
4. Interference phase: All participants were given 2 min for number crunching.
5. Final Recall Test: All participants completed the comprehension questionnaire individually, with no time limit.

The data that support the findings of this study are available on this link: https://osf.io/cxf8v/?view_only=e53405491228414bad47c55542b92112

### Results

**Figure 1:**
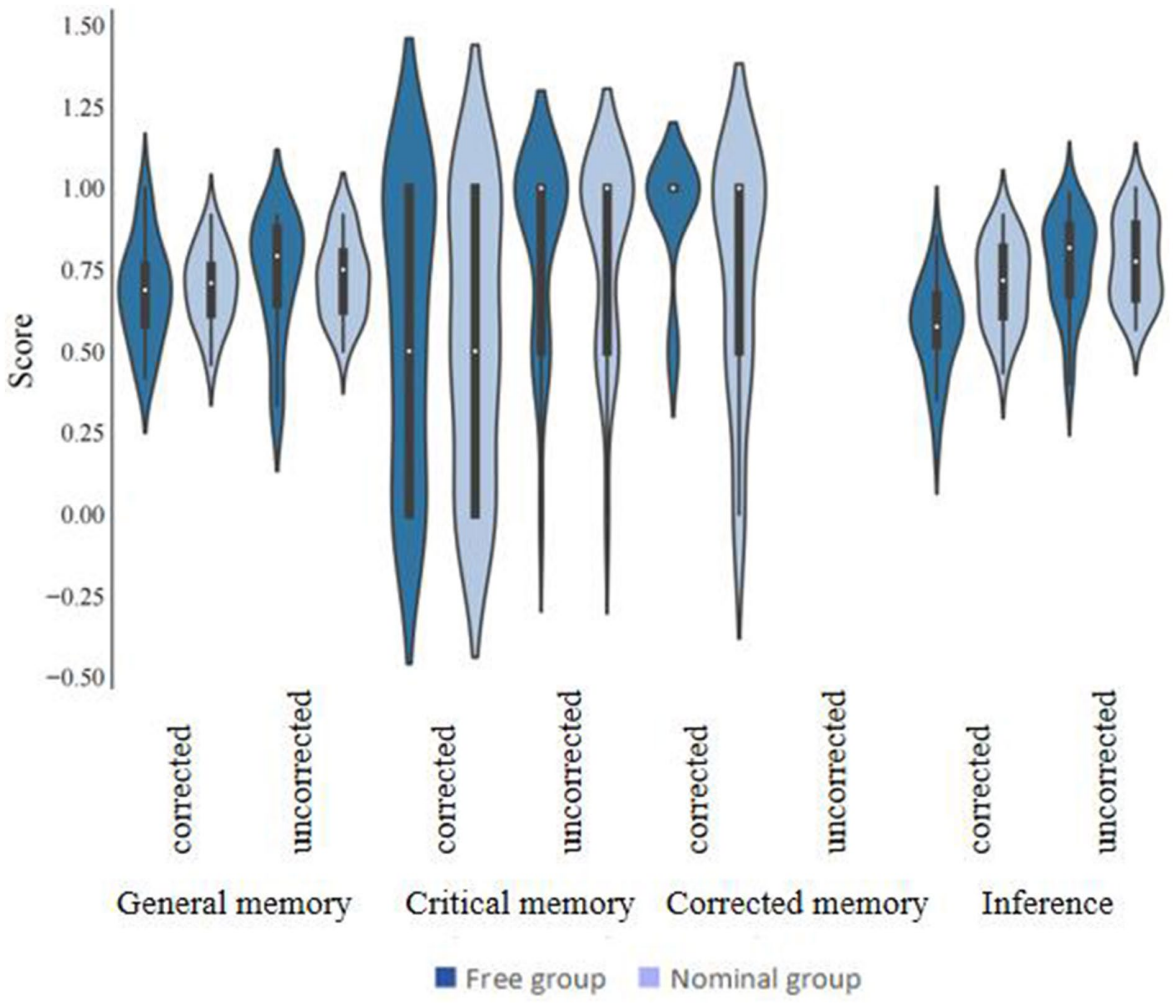
CIE scores in the free and nominal groups under different correction conditions (Experiment 1).

The statistical software SPSS 22.0 was utilized to perform descriptive statistics and conduct repeated measures analysis of variance (ANOVA) (Figure 1). All analytical procedures were based on *a priori* research hypotheses, and statistical significance was determined using a threshold of *p* < 0.05. All findings that reached statistical significance were duly reported. In Table 1, we compare the CIE scores and standard deviations for the free and nominal groups in Experiment 1 in the corrected condition versus the uncorrected condition, with the CIE scores being the General Memory Score, the Critical Memory Score, the Corrected Memory Score, and the Inference Scores, respectively. Separate repeated measures ANOVAs were conducted for each of the CIE scores, and the results were as follows:

**Table 1:** Mean (standard deviation) of CIE scores in the free and nominal groups under different correction conditions (Experiment 1).

	General memory		Critical memory		Corrected memory		Inference	
	Corrected	Uncorrected	Corrected	Uncorrected	Corrected	Uncorrected	Corrected	Uncorrected
Free group	0.68 (0.03)	0.77 (0.03)	0.58 (0.09)	0.83 (0.06)	0.92 (0.04)		0.57 (0.03)	0.70 (0.03)
Nominal group	0.71 (0.03)	0.72 (0.03)	0.54 (0.09)	0.81 (0.06)	0.73 (0.07)		0.71 (0.03)	0.78 (0.03)

#### General memory scores

There was no significant effect of the correction condition on general memory scores, *F*(1, 46) = 1.10, *p* = 0.30, ƞ^2^ = 0.02, 95%CI for ƞ^2^ = [0, 0.16]. There was no significant effect of the collaboration condition on general memory scores, *F*(1, 46) = 0.10, *p* = 0.76, ƞ^2^ = 0.00, 95%CI for ƞ^2^ = [0, 0.08]. The correction condition and collaboration condition interaction was not significant, *F*(1, 46) = 0.10, *p* = 0.76, ƞ^2^ = 0.00, 95%CI for ƞ^2^ = [0, 0.08].

#### Critical memory scores

The correction condition had a significant effect on critical memory scores, *F*(1, 46) = 14.26, *p* < 0.001, ƞ^2^ = 0.24, 95%CI for ƞ^2^ = [0.05, 0.42], and critical memory scores were significantly higher in the uncorrected condition than in the corrected condition. There was no significant effect of the collaboration condition on critical memory scores, *F*(1, 46) = 0.16, *p* = 0.69, ƞ^2^ = 0.00, 95%CI for ƞ^2^ = [0, 0.14]. There was no significant interaction between the correction condition and the collaboration condition, *F*(1, 46) = 0.02, *p* = 0.88, ƞ^2^ = 0, 95%CI for ƞ^2^ = [0, 0.02].

#### Corrected memory scores

The collaboration condition had a significant effect on corrected information memory scores, *t* (46) = 2.25, *p* = 0.03, Cohen’s d = 0.65, 95%CI for d = [0.07, 0.23], and the free collaboration group had significantly higher corrected memory scores than the nominal group.

#### Inference scores

The correction condition had a significant effect on Inference scores, *F*(1, 46) = 26.82, *p* < 0.001, ƞ^2^ = 0.27, 95%CI for ƞ^2^ = [0.15, 0.53], and Inference scores were significantly lower in the correction condition than in the no correction condition. The collaboration condition had a significant effect on Inference scores, *F*(1, 46) = 5.84, *p* = 0.02, ƞ^2^ = 0.11, 95%CI for ƞ^2^ = [0, 0.29], and the free collaboration group had significantly lower Inference scores than the nominal group. The Correction and Collaboration conditions interacted significantly, *F*(1, 46) = 5.13, *p* = 0.03, ƞ^2^ = 0.10, 95%CI for ƞ^2^ = [0, 0.28]. Simple effects tests illustrated that the Free Collaboration group’s Inference scores were significantly lower than those of the Nominal group in the Correction condition and that the Collaboration group’s Inference scores were not significantly different from those of the Nominal group in the incorrection condition.

### Discussion

The results of Experiment 1 found that free collaboration inhibited CIE. The free collaboration group remembered the corrected information significantly better than the nominal group, the inference of the free collaboration group relied more on later correction information rather than on the original misinformation.

Although experiment 1 supported the hypothesis of this study to a certain extent, the collaboration group in experiment 1 was limited to free collaboration, and it did not specifically explore whether different collaboration condition would have different impacts on CIE, and whether other factors in collaboration would have different impacts on CIE. Based on the above analysis, experiment 2, based on previous studies, divided collaboration modes into free collaboration and turn-taking group, and introduced the variable of recall time. According to the average recall time of subjects in experiment 1 was 12 min, the long recall time was set to 12 min, and the short recall time was set to 6 min, so as to explore whether different collaboration condition under different times would have an impact on CIE.

## Experiment 2

Although Experiment 1 supported the hypotheses of this study to a certain extent, it did not specifically explore whether different collaboration conditions would have different effects on CIE. Experiment 2 divides collaboration conditions into free collaboration and turn-taking collaboration and introduces the variable of recall time. Since the average recall time of participants in Experiment 1 was 12 min, the long recall time was set to 12 min and the short recall time was set to 6 min. The purpose of Experiment 2 is to determine whether different collaboration conditions at different times have an impact on CIE.

### Method

The Experiment design was a 2 (correction condition: corrected, uncorrected) × 2 (recall time: 6 min, 12 min) × 3 (collaboration condition: free group, turn-taking group, nominal group) mixed experimental design. The collaboration condition and recall time were between-participants factors, while the correction condition was a within-participants factor.

### Participant

An *a priori* power analysis in G*Power (Faul et al., 2007) suggested that we would need at least 54 participants to detect a high effect using ANOVA (*f* = 0.25; power = 0.95; *α* = 0.05). We therefore recruited 120 participants from an academic institution. As pre-registered, we removed those who failed the attention check (*n* = 4). This left a final sample of 116 participants (64 female, 52 males; Mage = 20.16, SDage = 1.58).

### Materials

The Experiment materials were the same as those used in Experiment 1.

### Procedure

**Figure 2:**
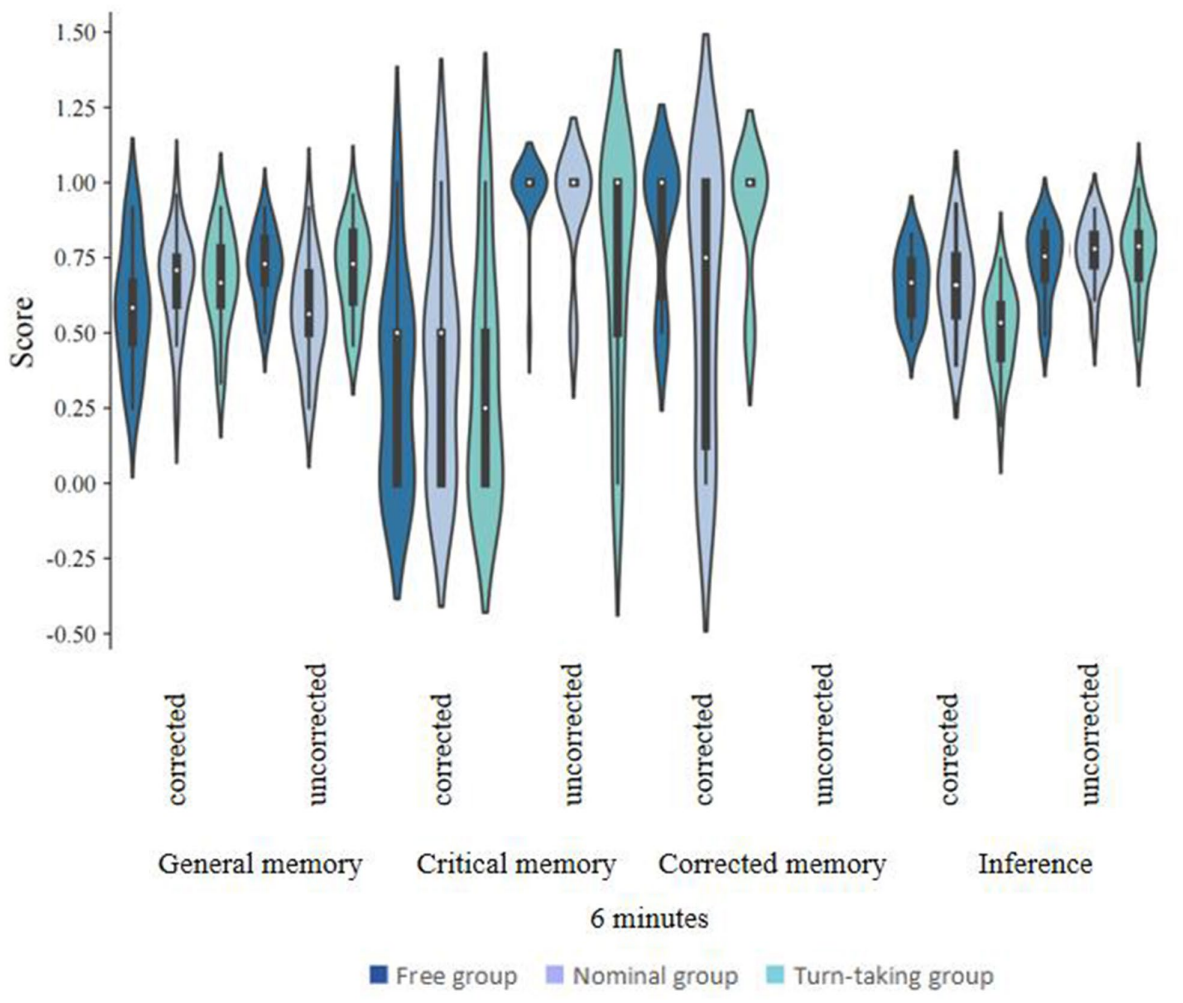
CIE scores for different collaboration conditions, recall time in different correction conditions under 6 min recall time (Experiment 2).

**Figure 3:**
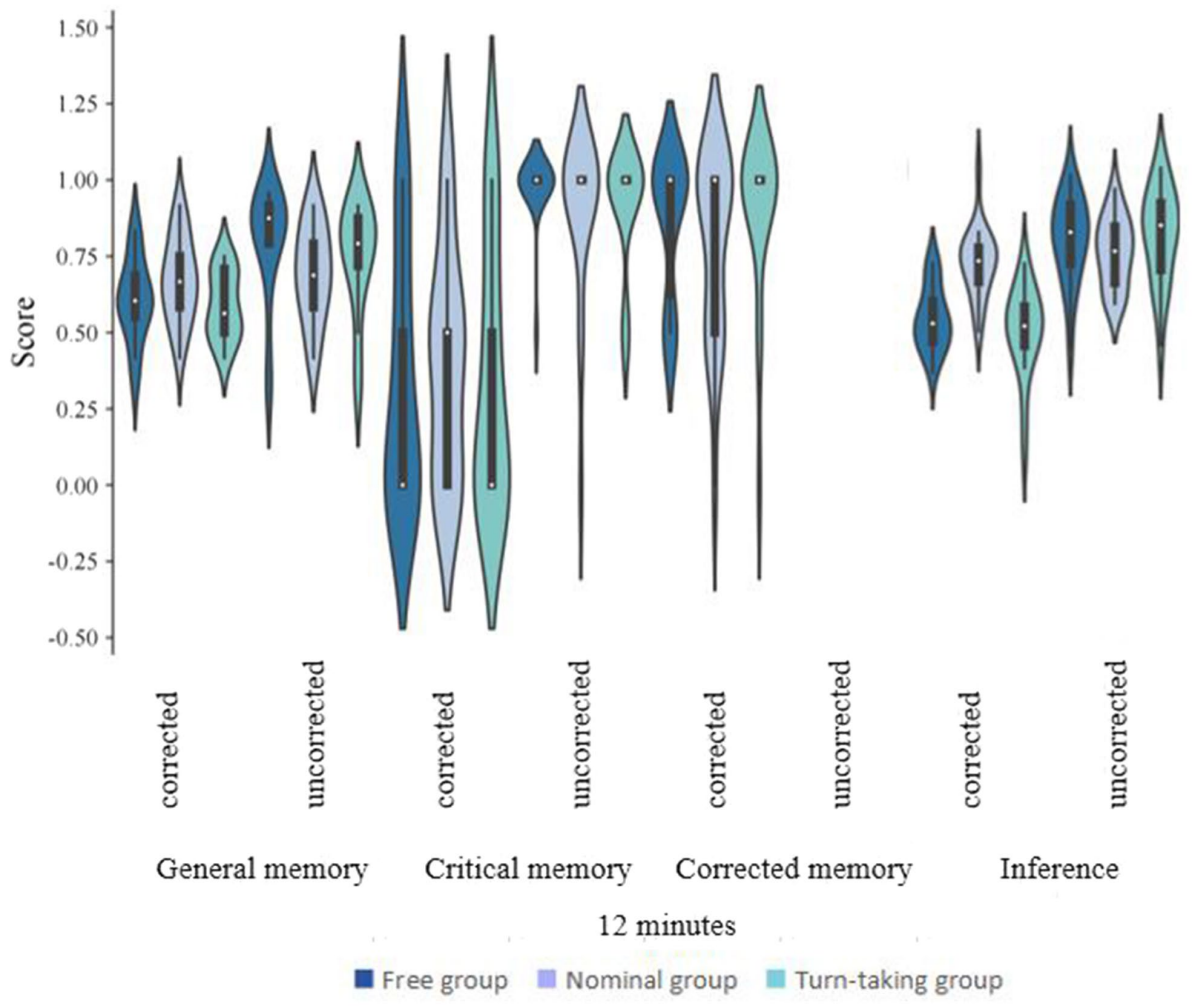
CIE scores for different collaboration conditions, recall time in different correction conditions under 12 min recall time (Experiment 2).

The study procedure was approximately the same as in Experiment 1, with the following differences (Figures 2, 3). Participants were randomly assigned to one of six study groups, with a recall time of 12 min for the group with the longer recall time and 6 min for the group with the shorter recall time. Each of the three members of the group took turn reporting one piece of information at a time. The information was both spoken and written on a piece of paper. If a participant could not recall the information within 30 s, they would move on to the next participant, and so on in rotation.

### Results

**Figure 4:**
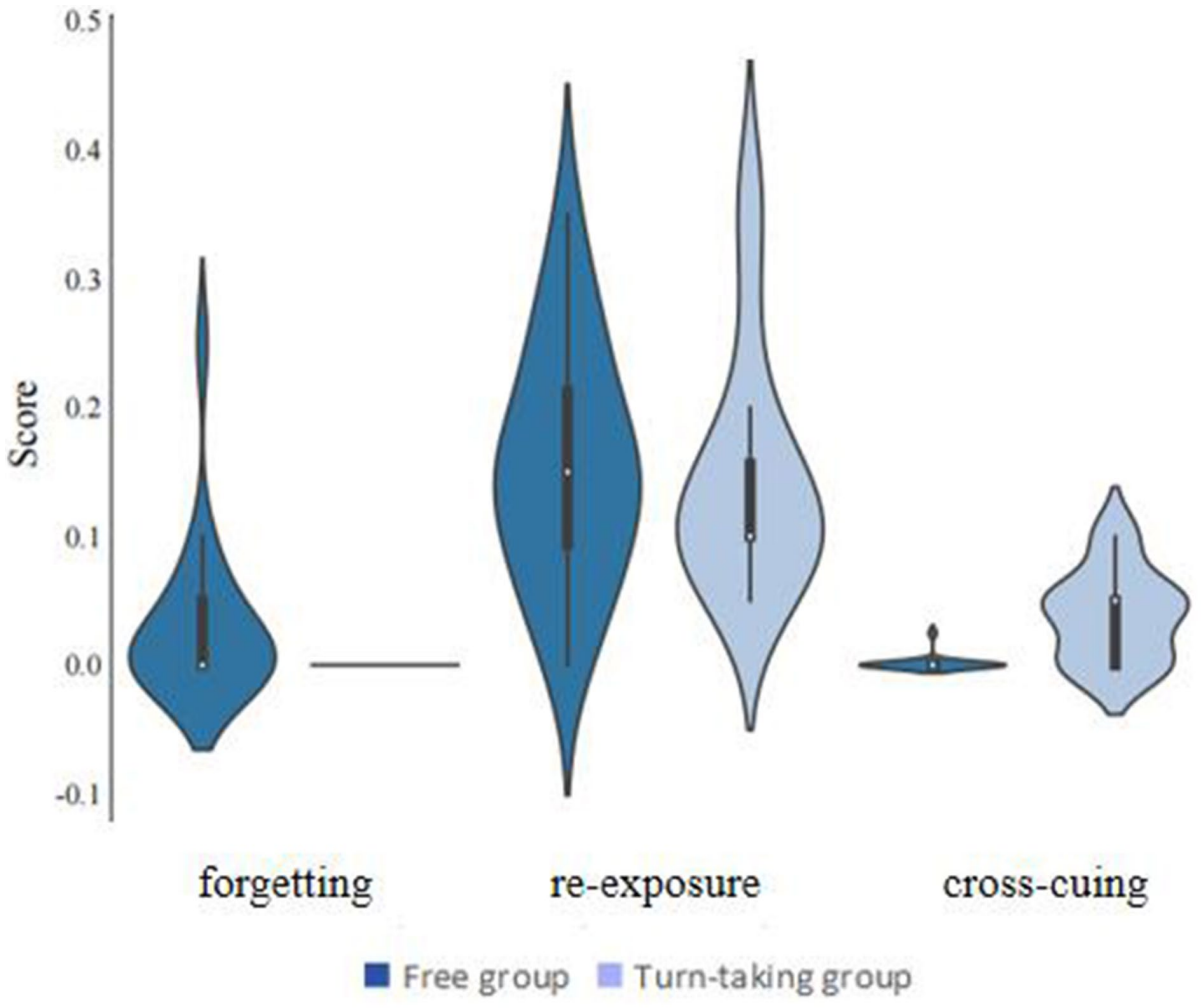
Memory and inference scores in the free and turn-taking groups (Experiment 3).

The statistical software SPSS 22.0 was utilized to perform descriptive statistics and conduct repeated measures analysis of variance (ANOVA) (Figure 4). All analytical procedures were based on *a priori* research hypotheses, and statistical significance was determined using a threshold of *p* < 0.05. All findings that reached statistical significance were duly reported. In Tables 2, 3, we compare the CIE scores and standard deviations of the free, turn-taking, and nominal groups in the corrected versus uncorrected condition for the conditions in Experiment 2 where the recall time was 6 and 12 min. Separate repeated-measures analyses of variance (ANOVAs) were conducted for each of the CIE scores, and the significant results were as follows:

**Table 2:** Mean (standard deviation) of CIE scores of free group, turn-taking group and nominal group under different correction conditions under 6 min recall time (Experiment 2).

	6 min							
	General memory		Critical memory		Corrected memory		Inference	
	Corrected	Uncorrected	Corrected	Uncorrected	Corrected	Uncorrected	Corrected	Uncorrected
Free group	0.58 (0.04)	0.72 (0.04)	0.37 (0.09)	0.97 (0.05)	0.87 (0.07)		0.66 (0.03)	0.740 (0.023)
Turn-taking group	0.67 (0.04)	0.57 (0.04)	0.39 (0.09)	0.92 (0.06)	0.61 (0.07)		0.66 (0.03)	0.77 (0.03)
Nominal group	0.65 (0.04)	0.72 (0.04)	0.30 (0.09)	0.75 (0.05)	0.88 (0.07)		0.51 (0.03)	0.76 (0.03)

**Table 3:** Mean (standard deviation) of CIE scores of free group, turn-taking group and nominal group under different correction conditions under 12 min recall time (Experiment 2).

	12 min							
	General memory		Critical memory		Corrected memory		Inference	
	Corrected	Uncorrected	Corrected	Uncorrected	Corrected	Uncorrected	Corrected	Uncorrected
Free group	0.61 (0.04)	0.80 (0.04)	0.33 (0.09)	0.97 (0.06)	0.86 (0.07)		0.52 (0.03)	0.78 (0.03)
Turn-taking group	0.67 (0.05)	0.68 (0.04)	0.40 (0.09)	0.88 (0.05)	0.78 (0.07)		0.70 (0.03)	0.72 (0.03)
Nominal group	0.60 (0.03)	0.75 (0.04)	0.36 (0.08)	0.93 (0.05)	0.88 (0.06)		0.48 (0.03)	0.79 (0.03)

#### General memory scores

There was a significant effect of the correction condition on general memory scores, *F*(1, 110) = 13.858, *p* < 0.001, ƞ^2^ = 0.112, 95%CI for ƞ^2^ = [0.03, 0.23], and general memory scores were significantly higher in the uncorrected condition than in the corrected condition. The collaboration condition had no significant effect on general memory scores, *F*(2, 110) = 1.00, *p* = 0.30, ƞ^2^ = 0.02, 95%CI for ƞ^2^ = [0, 0.08]; recall time had no significant effect on general memory scores, *F*(1, 110) = 2.74, *p* = 0.10, ƞ^2^ = 0.02, 95%CI for ƞ^2^ = [0, 0.11]. There was a significant interaction between the correction condition and the collaboration condition, *F*(2, 110) = 8.97, *p* < 0.001, ƞ^2^ = 0.14, 95%CI for ƞ^2^ = [0.04, 0.25], and inference scores were significantly lower for the free and turn-taking collaboration groups than for the nominal group in the correction condition. The correction condition and recall time interaction was not significant, *F*(1, 110) = 3.78, *p* = 0.06, ƞ^2^ = 0.03, 95%CI for ƞ^2^ = [0, 0.12]. The three-way interaction between the correction condition, the collaboration condition, and recall time was not significant, *F*(2, 110) = 0.13, *p* = 0.88, ƞ^2^ = 0, 95%CI for ƞ^2^ = [0, 0.29].

#### Critical memory scores

The correction condition had a significant effect on critical memory scores, *F*(1, 110) = 158.84, *p* < 0.001, ƞ^2^ = 0.59, 95%CI for ƞ^2^ = [0.47, 0.67], and critical memory scores were significantly higher in the no-correction condition than in the correction condition. There was no significant effect of the collaboration condition on critical memory scores, *F*(2, 110) = 1.42, *p* = 0.25, ƞ^2^ = 0.03, 95%CI for ƞ^2^ = [0, 0.10]. The recall time did not have a significant effect on critical memory scores, *F*(1, 110) = 0.49, *p* = 0.49, ƞ^2^ = 0, 95%CI for ƞ^2^ = [0, 0.06]. The correction condition and the collaboration condition interacted non-significantly, *F*(2, 110) = 0.79, *p* = 0.46, ƞ^2^ = 0.01, 95%CI for ƞ^2^ = [0, 0.07]. The correction condition and recall time The interaction was not significant, *F*(1, 110) = 0.16, *p* = 0.69, ƞ^2^ = 0, 95%CI for ƞ^2^ = [0, 0.05]. The three-way interaction of correction condition, collaboration condition, and recall time was not significant, *F*(2, 110) = 0.35, *p* = 0.71, ƞ^2^ = 0, 95%CI for ƞ^2^ = [0, 0.05].

#### Corrected memory scores

There was a significant effect of the collaboration condition on corrected memory scores, *F*(2, 110) = 4.87, *p* = 0.009, ƞ^2^ = 0.08, 95%CI for ƞ^2^ = [0.01, 0.18], and corrected memory scores were significantly higher in the free and turn-taking collaboration groups than in the nominal group, with a nonsignificant difference between the free and turn-taking collaboration groups. The recall time had no significant effect on corrected memory scores, *F*(1, 110) = 1.011, *p* = 0.32, ƞ^2^ = 0.009, 95%CI for ƞ^2^ = [0, 0.08]. The interaction between the correction and collaboration conditions was not significant, *F*(2, 110) = 1.03, *p* = 0.36, ƞ^2^ = 0.02, 95%CI for ƞ^2^ = [0, 0.08].

#### Inference scores

The correction condition had a significant effect on Inference scores, *F*(1, 110) = 117.01, *p* < 0.001, ƞ^2^ = 0.52, 95%CI for ƞ^2^ = [0.39, 0.61], and Inference scores were significantly higher in the no-correction condition than in the correction condition; the collaboration condition had a significant effect on Inference scores, *F*(2, 110) = 6.35, *p* = 0.002, ƞ^2^ = 0.10, 95%CI for ƞ^2^ = [0.02, 0.21]. Inference scores were significantly higher in the free collaboration and turn-taking collaboration than in the nominal group; The recall time had no significant effect on reasoning scores, *F*(1, 110) = 1.217, *p* = 0.27, ƞ^2^ = 0.01, 95%CI for ƞ^2^ = [0, 0.08]. The correction condition and recall time interaction was not significant, *F*(1, 110) = 2.24, *p* = 0.14, ƞ^2^ = 0.02, 95%CI for ƞ^2^ = [0, 0.10]. There was a significant interaction between the correction condition and the collaboration condition, *F*(2, 110) = 15.81, *p* < 0.001, ƞ^2^ = 0.22, ƞ^2^ = 0.22, 95%CI for ƞ^2^ = [0.10, 0.34]. Inference scores were significantly higher in the correction condition than in the nominal group in both the free collaboration and the turn-taking collaboration group; the three-way interaction of correction condition, collaboration condition, and recall time was significant, *F*(2, 110) = 5.81, *p* = 0.004, ƞ^2^ = 0.10, 95%CI for ƞ^2^ = [0.01, 0.20]. To understand the effect of collaboration style on CIE at different recall time, we did further analyses by conducting separate repeated-measures ANOVAs of CIE scores at different recall time and found that the results were different for the Inference scores. At a recall time of 6 min, the correction condition and the collaboration condition interacted significantly, *F*(2, 112) = 5.13, *p* = 0.03, ƞ^2^ = 0.1, 95%CI for ƞ^2^ = [0.01, 0.18]. Simple main effect tests illustrated that the Inference scores of the turn-taking collaboration group were significantly lower than the nominal versus the free collaboration group under the correction condition, and the free collaboration group was not significantly different from the nominal group; at a recall time of 12 min, the correction condition and collaboration condition interacted significantly, *F*(2, 112) = 16.23, *p* < 0.001, ƞ^2^ = 0.37 95%CI for ƞ^2^ = [0.01, 0.35]. A simple effects test illustrated that the Inference scores of the turn-taking collaboration group were significantly lower than those of the nominal group in the correction condition versus the free collaboration group and that there was no significant difference between the free collaboration and turn-taking collaboration groups.

### Discussion

At a recall interval of 6 min, the turn-taking group relied more on the corrected information for their inferences compared to both the free collaboration and nominal groups. No significant difference was observed between the free collaboration and nominal groups. At a recall interval of 12 min, both the turn-taking and free collaboration groups demonstrated greater reliance on the corrected information compared to the nominal group. However, no significant distinction was found between the turn-taking and free collaboration groups. These results indicate that the turn-taking group’s ability to inhibit the continued influence effect (CIE) was consistently effective, whereas the free collaboration group’s ability to inhibit CIE was influenced by recall time and lacked consistency.

## Experiment 3

Based on the results of Experiment 1 and Experiment 2, we hypothesized that the re-exposure and cross-cuing during the collaboration process may have influenced the Experiment outcomes. Experiment 3 aimed to investigate the differences between turn-taking collaboration and free collaboration in terms of re-exposure, cross-cuing, and information forgetting. This exploration aimed to shed light on the possible mechanisms underlying the differences between these two collaboration conditions.

In Experiment 3, the Experiment paradigm of “separate recall test, collaborative/separate recall test, separate recall test” was used. The baseline for memory results was established using the first test. The second memory results after the collaborative recall were then compared to the baseline. This was done to investigate whether the enhancement of memory in the different collaborative conditions was due to re-exposure and cross-cuing. And whether different collaborative conditions lead to forgetting.

### Method

The experimental design was a one-factor between-subjects design, with co-operation conditions divided into turn-taking and free collaboration groups. The dependent variables were re-exposure, cross-cuing, and forgetting scores were measured.

### Participants

An *a priori* power analysis in G*Power (Faul et al., 2007) suggested that we would need at least 34 participants to detect a high effect using ANOVA (*f* = 0.25; power = 0.95; *α* = 0.05). We therefore recruited 42 participants from an academic institution. As pre-registered, we removed those who failed the attention check (n = 2). This left a final sample of 40 participants (22 female, 18 males; Mage = 21.04, SDage = 1.28).

### Materials

#### Reading materials

In this Experiment, we attempted to investigate the mechanisms underlying the varying effects of collaborative conditions on recall and inference about corrected information. To achieve this, the participants were provided with reading materials that only contained the corrected version of the second piece in each scenario.

#### Recall test materials

A modified version of the questionnaire used by Xu et al. (2020) was employed, with the questionnaire reading “Write down what you can recall about XX scenarios.” If a participant reports a new item during the second individual recall, and this item was previously reported by other members of the group, it can be attributed to the role of re-exposure. If participants recalled new items during the second recall extraction that were not items that had previously appeared in reports by other members of the group, then this was attributed to the effect of cross-cuing. If an item recalled on the first personal memory test is not recalled on the second personal memory test, it is forgotten.

### Procedure

1. Learning phase: like Experiment 1, participants will read only the second corrected version of the article.
2. Interference phase: 2 min of data analysis.
3. Individual recall test phase: All participants completed the comprehension questionnaire individually, with the prompt “Please write down any information you can recall from the four scenarios” for an unlimited amount of time.
4. Interference phase: 2 min of data analysis.
5. Collaborative/individual recall test phase: Participants in the nominal and collaborative groups were informed that they could freely collaborate or recall information individually from the four scenarios for an unlimited amount of time.
6. Interference phase: 2 min of data analysis.
7. Individual recall test phase: All participants completed the comprehension questionnaire individually. There was no time limit.

### Results

The statistical software SPSS 22.0 was utilized to perform descriptive statistics and Independent t-test. All analytical procedures were based on *a priori* research hypotheses, and statistical significance was determined using a threshold of *p* < 0.05. All findings that reached statistical significance were duly reported. In Table 4, we compare the memory and inference scores of the free and turn-taking groups with their standard deviations for the Memory Scores, which are the Re-exposure Score, the Cross-cuing Score, and the Forgetting Score. Separate independent samples t-tests were conducted for each dependent variable, and the results were as follows:

**Table 4:** Mean (standard deviation) of memory and inference scores in the free and turn-taking groups (Experiment 3).

	Forgetting	Re-exposure	Cross-cuing
Free group	0.03 (0.01)	0.16 (0.02)	0.0 (0.01)
Turn-taking group	0.00 (0.00)	0.15 (0.02)	0.04 (0.01)

#### Forgetting scores

There was a significant difference in forgetting scores across collaboration conditions, *t* (38) = 2.24, *p* = 0.03, Cohen’s d = 0.71, 95%CI for d = [0.06, 1.34], with the free group having significantly higher forgetting scores than the turn-taking group.

#### Re-exposure scores

There was no significant difference in re-exposure scores across collaboration conditions, *t* (38) = 0.35, *p* = 0.73, Cohen’s d = 0.11, 95%CI for d = [−0.51, 0.73].

#### Cross-cuing scores

There was a significant difference in cross-cuing scores across collaboration conditions, *t* (38) = 4.75, *p* < 0.001, Cohen’s d = 1.50, 95%CI for d = [0.79, −2.20], with cross-cuing scores being significantly higher in the turn-taking collaboration group than in the free group.

### Discussion

The memory of the rotating group was affected by both the representation of others and the cross-cue, so the cooperative group showed stronger memory promotion and reasoning enhancement after collaboration. The memory of the free group was affected by the representation of others, and the content that was recalled separately for the first time was forgotten more, and the encoding intensity and familiarity of the corrected information were increased less, so the inhibition effect on CIE was poor.

## General discussion

The main purpose of this study was to investigate whether and how collaboration affects the continued influence effect of misinformation. It was found that the inhibit influence of collaboration on the continued influence effect of misinformation. Specifically, turn-taking group to inhibit CIE was stable, while the free group was affected by the recall time and could not consistently inhibit CIE. This was due to differences in memory for collaborative processes in terms of re-exposure and cross-cuing and information forgetting.

### The inhibit impact of collaboration on the CIE and its theoretical underpinnings

The Experiment results provide empirical support for the Knowledge Revision Theory. The results of this study found that collaboration led to an increase in the intensity of individuals’ encoding of corrective information. However, the intensity of encoding of original misinformation was not significantly different from that of the nominal group. This suggests that in the collaborative condition, corrective information gained the final output in the competition for activation with original misinformation. It also indicates that corrective information could be activated, effectively inhibiting the continued influence effect of misinformation. The reason why corrective information was able to dominate the entire information network under the collaboration condition was due to the re-exposure and cross-cuing that occurred during the collaboration process. Cross-cuing elicits additional or repeated recall, while re-exposure facilitates repeated learning. This process provides individuals with effective cues for recalling information, thereby enhancing the strength of memory encoding (Basden et al., 2000). Collaboration allows group members to reinforce corrections through re-exposure and cross-cuing. As individuals become more familiar with the corrected information, their encoding strength improves, leading to better memory retention of the corrected information and increased influence of the corrected information on inference-making.

### The impact of different collaboration conditions on the CIE and its internal mechanism

The findings of the present Experiment demonstrate that collaborative conditions influence CIE, turn-taking collaboration stably inhibits CIE at different recall time, and the inhibitory effect of free collaboration on CIE is affected by recall time; when the recall time is longer, free collaboration inhibits CIE. However, when the recall time is shorter, free collaboration does not inhibit CIE. The effect of different collaboration conditions on the continued influence effect of misinformation is moderated by recall time, with the intrinsic mechanisms being the re-exposure, cross-cueing, and forgetting during the collaboration process. Specifically, the group engaged in turn-taking collaboration shows that their memories were influenced by re-exposure and cross-cuing, whereas the group engaged in free collaboration showed that their memories were influenced only by re-exposure.

According to Knowledge Revision Theory, the magnitude of CIE partly depends on the strength of memory, which is primarily influenced by the familiarity of the information and the frequency of its repetition. A previous study has demonstrated that cross-cuing elicits additional or repeated recall, while re-exposure elicits repeated learning. It has also been found that repeated recall produces more long-term benefits for subsequent individual recall than repeated learning (Eghbaria-Ghanamah et al., 2021), so turn-taking collaboration stably inhibit CIE across recall time. Additionally, the present study found that free collaboration was primarily influenced by re-exposure. Re-exposure counteracted the collaborative recall interruptions caused by retrieval strategies (Blumen et al., 2014). This means that members of a free collaboration can overcome past retrieval strategy interruptions after the collaboration process, and their memory potential is restored to a normal level (Marion and Thorley, 2016; Nie et al., 2023). Furthermore, when collaborative recollections are prolonged, the reappearance of others has a longer-lasting impact on memory, offsetting more costs. The potential benefits of retrieval strategy disruption include the ability to overcome past disruptions, restore memory to normal levels, and more repetitions of the corrected information result in subjects remembering the corrected information more intensely, thus free collaboration inhibit CIE. When collaborative recall was short, others reproduced for a shorter period, resulting in less cost incurred to offset retrieval strategy interruptions. Additionally, the memory potential had not been fully restored, and the number of repetitions of the corrected information is less, and free collaboration is not able to inhibit CIE.

### The impact of recall time on the CIE and its internal mechanism

It was found that the alternating group inhibited CIE stably, while the free group was affected by recall time. When the recall time is 6 min, the reasoning of the rotating cooperative group is more dependent on the later corrected information than that of the free cooperative group and the nominal cooperative group. There is no significant difference between the nominal cooperative group and the free cooperative group. However, when the recall time is 12 min, the reasoning of the rotating cooperative group and the free cooperative group is more dependent on the later corrected information than that of the nominal group. There was no significant difference between the rotating collaboration group and the free collaboration group, which indicated that the phenomenon of rotating collaboration inhibiting the continuous influence of false information was stable, but the free collaboration was not stable, and only when individuals fully cooperated with others and shared memories, the free collaboration could inhibit CIE. The reasons for the different results of free collaboration and alternate collaboration under different recall time conditions are as follows: based on the previous theories of others’ reproduction and cross-cues, and the discovery that collaboration can destroy individual memory strategies to a certain extent and lead to individual forgetting, experiment 3 of this study was carried out.

### Limitations and future research

This study has several limitations. First, while exploring the effect of collaboration on the continued influence effect (CIE), individual differences among participants can lead to variability in the collaboration process. Controlling for all potential variables in the collaborative setting is challenging, and these uncontrolled factors may influence the results. Additionally, the study’s duration was quite lengthy, which may have led to participant fatigue. This fatigue could have impacted the outcomes, particularly during the final memory test session. Consequently, participants might have provided fewer responses to the free recall questions in the final test due to decreased engagement.

In the future, we can further investigate the impact of collaboration on CIE. Specifically, we can examine how collaboration affects CIE under various influencing factors. Previous studies have found that collaboration on memory is influenced by various factors such as group sizes, intervals, the nature of reading materials, and individual differences in participants. Future studies can further investigate the impact of collaboration on collective intelligence enhancement, considering these aforementioned factors. The research design of this study combines the collaborative memory paradigm with the CIE paradigm. In future studies, researchers can further innovate the study design, streamline the procedures, minimize the impact of participant fatigue and guessing on the results, and effectively manipulate the collaborative variables to investigate their effects on CIE. In addition, future research can further explore the neural mechanisms through which collaboration affects CIE. In recent years, several studies have started to investigate the neural mechanisms behind the persistent effects of misinformation (Hua et al., 2022). One study found that the activation of the left middle temporal gyrus was significantly weaker in the correction condition during the encoding phase compared to the control condition. Additionally, the activation of the frontal gyrus and the anterior cingulate gyrus was weaker in the correction condition during the extraction phase. Furthermore, the functional connectivity between the frontal gyrus and the precentral gyrus was stronger. However, it remains unknown whether collaboration influences the activated brain regions of the CIE. However, it is not yet known whether collaboration affects the activation of brain regions in CIE. Future studies may explore the neural mechanisms underlying the effects of collaboration on CIE.

## Data Availability

The datasets presented in this study can be found in online repositories. The names of the repository/repositories and accession number(s) can be found in the article/Supplementary material.
