# Supplementary material for: The inhibitory impact of collaboration on the continued influence effect of misinformation
Source: Front Psychol. 2024 Oct 25;15:1487146. doi: 10.3389/fpsyg.2024.1487146 (PMC11549669; doi:10.3389/fpsyg.2024.1487146)
Supplement: Supplementary file 1 [file Table_1.DOCX]

**Appendix** (using the bushfire scene as an example):

**1.Reading material**

**Article 1**: There is a forest fire in the north-east of Victoria that suddenly got out of control overnight and firefighters have been fighting it. The bushfire is close to homes in the town of Oroa and is currently in a very dangerous situation, but there has been no damage to property. David from the National Fire Service (CFA) says authorities are investigating the cause of the fire and early evidence suggests that it was deliberately lit. Until this morning, emergency services were working tirelessly to extinguish the blaze, which should now be unlikely to pose any further threat to the local community.

**Uncorrection**: Firefighters have managed to contain a bushfire in north-east Victoria after a full day's work. There are no reports of injuries or property damage, with most of the land loss occurring in rural fringe areas and nearby forest reserves. The suspected burn area is about 50,000 hectares. Amy, a local resident, said she was relieved that no one was harmed by the fires. Nonetheless, she believes that community members should be prepared for a recurrence of the disaster.

**Correction**: After a thorough investigation and review of witness reports, authorities have concluded that the fire was caused by a lightning strike. The rest of the conditions are the same as without correction.

**2. Questionnaire**

1. Describe the 'bushfire' scenario. 2.

2. Where did the bushfires occur?

a. California b. Mississippi c. New York d. Victoria

3. Why was Amy, a local resident, relieved?

a. no one was hurt b. her house was not affected

c. her pets survived d. it started to rain

4.How many hectares of jungle were burned?

a.100000 b.500000 c.50000 d.200000

5. the cause of the jungle fire mentioned in the text?

6.How much do the local people distrust the National Fire Service after the fires? (1-10 from very trusting trusting to very distrusting)

1 2 3 4 5 6 7 8 9 10

7. should anyone be punished for the forest fire? (1-10 from not at all to completely)

1 2 3 4 5 6 7 8 9 10

8.The government should put more resources into preventing human arson. (1-10 from strongly disagree to strongly agree)

1 2 3 4 5 6 7 8 9 10

**3. primary and secondary information**

**Primary Message.**

CFA battled the bushfires.

There was no property damage.

**Secondary Message.**

Firefighters still working to extinguish/no further threats/bushfires under control.

Damage to rural fringe areas and forest reserves.
